# Multilocus sequence typing provides insights into the population structure and evolutionary potential of *Brenneria goodwinii*, associated with acute oak decline

**DOI:** 10.1371/journal.pone.0178390

**Published:** 2017-06-01

**Authors:** Maciej Kaczmarek, Martin S. Mullett, James E. McDonald, Sandra Denman

**Affiliations:** 1 Bangor University, School of Biological Sciences, Bangor, Gwynedd, United Kingdom; 2 Forest Research, Centre for Ecosystems, Society and Biosecurity, Alice Holt Lodge, Farnham, Surrey, United Kingdom; Beijing Institute of Microbiology and Epidemiology, CHINA

## Abstract

*Brenneria goodwinii* is one of the most frequently isolated Gram-negative bacteria from native oak species, *Quercus robur* and *Q*. *petraea*, affected by acute oak decline (AOD) in the UK. We investigated the population biology of this bacterial species using a multilocus sequence analysis to determine the population structure and evolutionary potential. Seven partial housekeeping genes were used in the analyses. Amongst 44 bacterial strains from seven different locations, we identified 22 unique sequence types [STs]; only one ST was found at two separate locations. Phylogenetic and cluster-based analyses suggested that *B*. *goodwinii* STs form two main distinct groups; however, no geographical pattern of their distribution could be observed. Clonality and recombination tests demonstrated that the studied population is primarily clonal, however both mutation and recombination processes play a role in shaping the genetic structure and evolution of the population. Our study suggests that the *B*. *goodwinii* population on oak in the UK has an endemic form, with background recombination appearing to generate new alleles more frequently than mutation, despite the introduction of nucleotide substitutions being approximately twice less likely than mutation. The newly emerged STs subsequently undergo clonal expansion to become dominant genotypes within their specific geographical locations and even within the individual host oak trees.

## Introduction

In recent years, an increasing number of episodes of a serious rapid decline of native pedunculate oak (*Quercus robur*) and sessile oak (*Q*. *petraea*) in the UK have been reported [1]. Declines are considered a separate category of tree diseases as they appear to result from interactions between a combination of biotic and abiotic factors [2, 3]. Currently, two forms of this disorder are recognised in Britain: acute oak decline (AOD) and chronic oak decline (COD) [4]. Both forms of decline are usually episodic and with a complex cause, but certain agents may play a dominant role [5]. Acute oak decline (AOD) is a relatively new and emerging complex disease syndrome that occurs on native oak in the UK and continental Europe [6]. In more severe cases, the decline may lead to death of the affected tree within 4–5 years of the initial onset of the disease. Typical symptoms of AOD include stem bleeding from longitudinal cracks in the bark and tissue necrosis located beneath the weeping patches. D-shaped exit holes and larval galleries of the two spotted oak buprestid beetle *Agrilus biguttatus* (TSOB) have also been associated with AOD [7]. A plethora of bacterial species have so far been identified within lesions on affected trees. *Gibbsiella quercinecans* and *Brenneria goodwinii* remain the most consistently isolated microorganisms from oak tissues expressing typical symptoms of AOD in Britain [8, 9], with *B*. *goodwinii* being frequently and consistently detected using metabarcoding methods [10]. Both species were initially assigned to *Enterobacteriaceae* [11, 8]. Most recently however, this family has been re-evaluated and, subsequently, *B*. *goodwinii* has been placed in a new family *Pectobacteriaceae* [12].

When delineating units of biological diversity by linking genetic diversity with ecology and evolutionary theory, it became apparent that approaches that can provide high resolution are needed to make sequence-based phylogenetics and population biology easier to interpret [13, 14, 15]. Multilocus sequence analysis (MLSA) offers considerable advantages, mainly due to its high reproducibility and reliability thus allowing the generation of cumulative global databases [16] and provides a very meaningful prediction of whole-genome relatedness with a level of resolution comparable to DNA-DNA hybridization [17, 18, 19]. Not only has this approach been widely applied to unravel the taxonomic relationships in a series of genera [20, 21, 22, 23, 24, 25], it has also been applied in the investigations of intraspecific diversity such as ecovars [26, 27]. The use of multiple genes for typing has a number of advantages. Similar to MLSA, the gene targets for multilocus sequence typing (MLST) are housekeeping genes which encode for proteins that are essential for cellular function, therefore the selection pressures acting upon them are thought act uniformly across individuals in a particular environment. Combined analyses of multiple sites on the genome also provide increased informative characters and better discrimination than a single gene could provide, thus overcoming the effect of recombination events that could have occurred at a single locus [16, 28, 29].

To date, *B*. *goodwinii* population structure in the UK and beyond has not been studied. As *B*. *goodwinii* is consistently associated with AOD stem lesions and has been shown to have necrogenic capability on oak [6], there is a need to gain insights into the structure of the population so that epidemiological inferences can be made. Therefore, a reliable and robust population genetics method is required to gain initial insights into intraspecific diversity of this AOD-associated bacterium, and to identify different clones if present, with significant confidence. It is also useful to consider fundamental and applied aspects such as distribution patterns, potential spread of specific clonal lineages and persistence of genotypes or established clonal lineages in the UK, as these may provide insights into their role as a putative contributing factor leading to the observed increase in AOD reports on native oak. Such data could become useful in the prediction of disease spread in the future. In this study, a reliable MLST scheme was developed to analyse the structure, diversity and evolutionary potential of *B*. *goodwinii* populations through investigating to what extent the intraspecific diversity of this species correlated with the prevalence of a particular clone and/or set of clones in the UK. In this manuscript, the application of MLST and the resulting phylogeny used for dissecting a poorly understood natural intraspecific diversity and distribution of this common AOD-associated bacterium is discussed.

## Materials and methods

### Isolation of *B*. *goodwinii* strains

**Fig 1:**
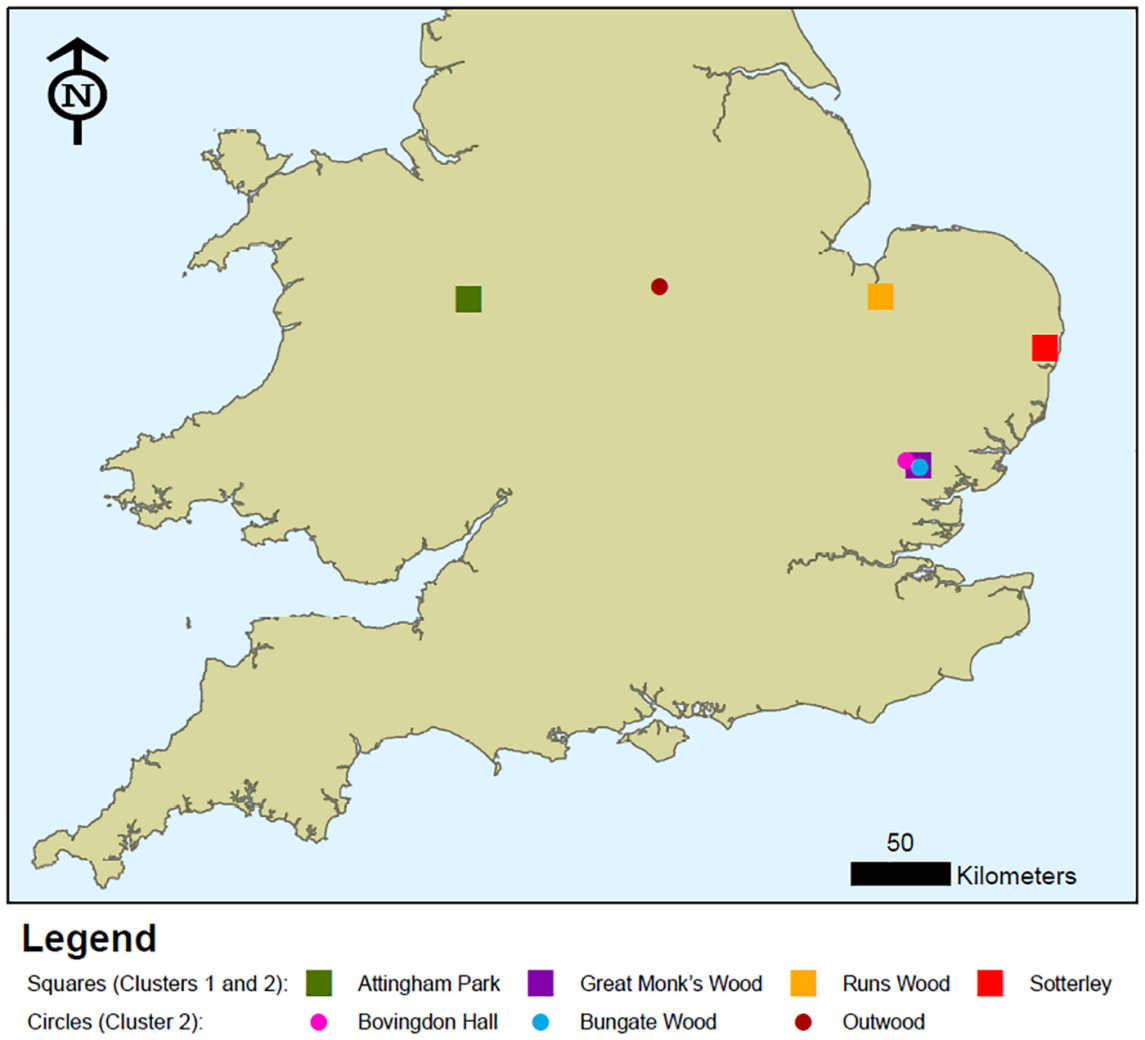
An ArcGIS map indicating the location of AOD-affected sites in England, sampled in this study. The shape indicates the distribution of the ancestral clusters 1 and 2, i.e. square = both clusters, circle = cluster 2 only as estimated by maximum likelihood method and STRUCTURE analysis (Figs 3 and 5, respectively). Green background indicate woodland areas.

*B*. *goodwinii* strains were obtained from AOD affected trees on six geographically separated sites (Fig 1). From Attingham Park (N 52°41’5” W 2°40’6”), 7 strains were obtained from a single tree; from Bovingdon Hall (N 51°55’31” E 0°33’13”)—4 strains from 2 trees; Bungate Wood (N 51°53’30” E 0°39’23”)—5 strains from one tree; from Great Monks Wood (N 51°53’47” E 0°38’42”) and Runs Wood (N 52°40’15” E 0°24’44”)—10 strains from 3 trees per site; and at Sotterley (N 52°24’34” E 1°36’55”)—5 strains from a single tree, but an additional strain from a Turkey oak (*Q*. *cerris*) with AOD symptoms was included. Due to the destructive nature of sampling procedure, the number of trees/ lesions sampled per location was limited and dependent on receiving direct permission from landowners/ park officers and thus, there is an unequal representation in terms of number of strains per site. A positive control strain, the Type of *B*. *goodwinii* obtained from Outwood (N 52°44’21” W 1°14’21”) was also included (Table 1).

**Table 1:** List of isolates and their origin. All the listed trees are pedunculate oak, *Q*. *robur*, except one Turkey oak, *Q*. *cerris*, as indicated in the table.

Strain	Tree number	Location within tissue	Site
**RW130**	Tree 4	Symptomatic inner bark	Runs Wood
**RW142b**	Tree 4	Symptomatic sapwood	
**RW85b**	Tree 3	Symptomatic inner bark	
**RW74c**	Tree 3	Symptomatic outer bark	
**RW73a**	Tree 3	Symptomatic outer bark	
**RW105a**	Tree 3	Symptomatic heartwood	
**RW45b**	Tree 2	Symptomatic sapwood	
**RW53b**	Tree 2	Symptomatic heartwood	
**RW37a**	Tree 2	Symptomatic inner bark	
**RW62**	Tree 2	TSOB gallery	
**GM11b**	Tree 1	Symptomatic inner bark	Great Monk’s Wood
**GM21b**	Tree 1	Symptomatic inner bark	
**GM26b**	Tree 1	Symptomatic sapwood	
**GM29c**	Tree 1	Symptomatic sapwood	
**GM33b**	Tree 1	Symptomatic sapwood	
**GM40b**	Tree 1	TSOB gallery	
**GM82b**	Tree 3	Non-symptomatic heartwood	
**GM61b**	Tree 2	Symptomatic sapwood	
**GM62b**	Tree 2	Non-symptomatic heartwood underlying lesion	
**GM59**	Tree 2	Symptomatic inner bark	
**BH1-63**	Tree 1	Symptomatic inner bark	Bovingdon Hall
**BH4-23**	Tree 4	TSOB gallery	
**BH4-13**	Tree 4	TSOB gallery	
**BH4-24**	Tree 4	TSOB gallery	
**SOT2-26**	Tree 2	Inner bark TSOB pupal chamber lesion	Sotterley
**SOT2-27a**	Tree 2	Inner bark TSOB pupal chamber lesion	
**SOT2-29a**	Tree 2	TSOB gallery	
**SOT2-34a**	Tree 2	TSOB gallery	
**SOT2-28a**	Tree 2	Inner bark pupal chamber lesion	
**SOT3-4a**	Turkey oak	Symptomatic inner bark	
**BW2-8**	Tree 2	Symptomatic inner bark	Bungate Wood
**BW2-18**	Tree 2	Symptomatic sapwood	
**BW2-17**	Tree 2	Symptomatic sapwood	
**BW2-24**	Tree 2	Symptomatic sapwood	
**BW2-31**	Tree 2	Symptomatic sapwood	
**BW1-7**	Tree1	TSOB gallery	
**AT1-1a**	Tree 1	Lesion 1 Symptomatic inner bark	Attingham Park
**AT1-2a**	Tree 1	Lesion 2 Symptomatic inner bark	
**AT1-2f**	Tree 1	Lesion 2 Symptomatic inner bark	
**AT1-1b**	Tree 1	Lesion 1 Symptomatic inner bark	
**AT1-1g**	Tree 1	Lesion 1 Symptomatic inner bark	
**AT1-3g**	Tree 1	TSOB gallery	
**AT1-3h**	Tree 1	TSOB gallery	
**FRB141 (T)**	Tree1	Lesion on buttress root	Outwood

Strains were isolated from the lesion margins present on the oak bark panels in years 2012–2013, whereas Attingham Park strains were obtained in 2016. Some strains were also isolated from apparently unaffected tissue of oak trees that exhibited symptoms of AOD. Isolates were subsequently stored as pure colonies in glycerol stock at -80°C.

### DNA extraction procedure

Bacterial strains were cultured in 5 ml Luria Broth (Miller’s LB broth) (LB) medium (Sigma-Aldrich 3050 Spruce Street, ST. Louis, MO 63103 USA) overnight at 28°C. The strains used for inoculating LB broth came from previously generated pure single colony strains that underwent multiple subculturing procedure from initial isolation plates to ensure only single genotypes are used for DNA extractions. Cells were harvested by centrifugation at 10000 × g for 3 minutes, washed by resuspending in 1.5 ml sterile distilled water (SDW), and centrifuged again for 3 minutes at 10000 × g. Two additional wash steps were carried out to remove all remaining LB medium from the harvested cells. DNA extraction was carried out using FastDNA Spin Kit (MP Biomedicals LLC, Solon, OH, USA) following the manufacturer’s instructions.

### *B*. *goodwinii* identification assay

In order to reaffirm the identity of the *B*. *goodwinii* strains, PCR amplification of the gyrB gene was performed using the method and primers previously described for *Pantoea* species [30]. The PCR amplification products were purified using a MinElute 96 UF PCR Purification Kit (Qiagen GmbH, 40724 Hilden, Germany), according to the manufacturer’s instructions. The samples were then sequenced (Sanger sequencing) at Source BioScience (William James House, Cowley Road, Cambridge CB4 0WU, UK).

### *B*. *goodwinii* MLST scheme

To develop and evaluate the MLST method for determining the population structure of *B*. *goodwinii*, ten partial gene sequences (LSU (26S), SSU (16S), rpoB, atpD, dnaJ, dnaN, infB, nusA, gyrB and abc) were obtained from a set of 44 field strains (Table 1), isolated from oak trees that had expressed symptoms of AOD, using newly developed sets of *B*. *goodwinii*-gene specific primers (Table 2). Primers were designed against the individual genes from the whole genome sequence of *B*. *goodwinii* FRB141(T) (James Doonan 2016, PhD Thesis, Bangor University). The *B*. *goodwinii* type strain FRB141 from Outwood, Loughborough, UK [8] was also added to the analysis as a reference isolate. Seven partial genes (rpoB, gyrB, dnaJ, dnaN, infB, nusA and abc) were chosen to establish the MLST scheme for population genetic studies on this bacterium. The choice was based on the presence of polymorphic sites detected within the genes, and thus, their ability to better discriminate between different sequence types (Table 3). Since 26S, 16S and atpD did were identical, they were not included in the analyses.

**Table 2:** List of MLST primers used in this study. 5’- 3’oligonucleotide sequences are shown.

Forward primer	Primer sequence	Reverse primer	Primer sequence
**BG_rpoB_F**	tgaaacgtctgctggatctg	BG_rpoB_R	cccaaaatgcggtaacaagt
**BG_dnaJ_F**	cggatcgtaatccaggtgat	BG_dnaJ_R	ctttgccgatcgttcagact
**BG_dnaN_F**	gttgcaggttaccgaaggag	BG_dnaN_R	atcggcataacgacataggc
**BG_gyrB_F**	tcgcgaaggtaaggttcatc	BG_gyrB_R	cacttcctgggaagagagca
**BG_infB_F**	ctgaaccgagatcgtggaat	BG_infB_R	ttctcacgacgcagaatgac
**BG_nusA_F**	cccttgatgttggcgattac	BG_nusA_R	ccttagctcgttcacgcaat
**BG_abc_F**	ctgagactgtccgtcgtgtg	BG_abc_R	acgctgagccctgaaataga

**Table 3:** Characteristics of the seven genes used for the analysis of *B*. *goodwinii* populations.

Gene fragment	m	n	S	Mean % GC	p_s_	Θ	π	D	dN/dS
**abc**	44	907	24	56.34	0.026461	0.006083	0.010028	2.153692^a^	0.0652
**dnaJ**	44	808	6	56.84	0.007335	0.001686	0.001746	0.092503^b^	0.0705
**dnaN**	44	909	17	56.02	0.018702	0.004299	0.006476	1.614958^b^	0.0161
**gyrB**	44	643	11	53.74	0.017107	0.003954	0.005167	0.918184^b^	0.0000
**infB**	44	912	3	53.75	0.003289	0.000756	0.000838	0.230376^b^	0.0000
**nusA**	44	917	7	52.81	0.007634	0.001755	0.002231	0.735866^b^	0.0000
**rpoB**	44	901	14	55.10	0.015538	0.003572	0.004794	1.061058^b^	0.0000
**concatenated**	44	5997	82	54.94	0.013651	0.003138	0.004474	1.528550	-

*Abbreviations*: *m* = number of sequences, *n* = total number of bp, *S* = Number of segregating sites, *Mean % GC* = average percentage of GC nucleotide base pairs in the gene sequences, *p*_s_ = *S*/*n*, *Θ* = *p*_s_/a_1_, *π* = nucleotide diversity, *D*—the Tajima test statistic;

^a^–significantly different from 0,

^b^—insignificant difference; dN/dS = ratio of non-synonymous (dN) to synonymous (dS) substitutions.

### PCR amplification

Conventional PCR amplification was performed using a GeneAmp PCR System 9700 (Perkin Elmer Applied Biosystems, Norwalk CT., 06859 USA). DNA fragments for individual genes were amplified separately in 25 μl reactions with GoTaq Green Master Mix (Promega UK, Delta House, Southampton Science Park, Southampton SO16 7NS, UK) at the uniform prescribed temperature conditions for all primer sets added at the concentration of 0.25 μM. Genomic DNA was standardised to 5 ng × μl^-1^, denatured for 5 minutes at 94°C, followed by 30 cycles each consisting of 30 s at 94°C, 30 s at 58°C and 60 s at 72°C with a final extension step at 72°C for 7 min. The same reaction conditions were used to amplify all the genes. Gel electrophoresis of PCR products was carried out on a 1% agarose gel and visualised using Biorad Gel Doc 1000. Each gene fragment was purified using MinElute 96 UF PCR Purification Kit (Qiagen GmbH, 40724 Hilden, Germany), according to the manufacturer’s instructions. Purified DNA samples were then sequenced as previously described for gyrB identification assay.

### Sequence analysis

Geneious v8.1.4 (Biomatters Ltd.) [31] was used to generate pairwise sequence alignments for individual housekeeping genes and, subsequently, for the concatenation of the seven gene sequences of each isolate to generate unique multilocus sequences for subsequent phylogenetic analysis. Data were checked for quality, and for each gene, both ends of the sequence were trimmed to the same length prior to concatenating the sequences. Geneious 8.1.4 was used to obtain the range of intraspecific sequence similarity (%) for each gene.

MEGA v6.06 software [32] was used to estimate sequence diversity and statistical values that included the number of segregating sites (*S*), Tajima’s pi (*π*) as a measure of nucleotide diversity [33], the average number of pairwise nucleotide differences (*p*_s_) [33], and Watterson’s theta (*Θ*)–for individual genes as well as the concatenated 7-gene sequence [34]. Tajima’s D was used to assess whether sequence variation in each gene for all isolates deviated from neutrality [35].

START2 [36] was used to determine the average percentage GC (guanine + cytosine) content for each gene fragment. The ratio of non-synonymous to synonymous substitutions (dN/dS) at each locus was also calculated.

### Phylogenetic analysis using maximum likelihood (ML) method

Selection of the best-fit model for nucleotide substitution patterns at each protein-coding locus was performed using the MEGA6 model test tool. The best nucleotide substitution model for the corresponding concatenated sequences, based on the lowest Bayesian Information Criterion (BIC) and Akaike Information Criterion (AIC) values, was selected to generate the ML phylogenetic tree. For each model, the Maximum Likelihood value (lnL) and the number of parameters (including branch lengths) were also estimated. Non-uniformity of evolutionary rates among sites was modelled using a discrete Gamma distribution (+G) with 5 rate categories, and assuming that a certain fraction of sites are evolutionarily invariable (+I). Whenever applicable estimates of the gamma shape parameter, the estimated fraction of invariant sites (I), and assumed or estimated values of transition/transversion bias (R), were also used for phylogenetic analysis. For estimating ML values, a tree topology was automatically computed. All positions containing gaps and missing data were eliminated. For the alignment of concatenated sequence data of all 44 strains of *B*. *goodwinii*, the Hasegawa-Kishino-Yano substitution model (HKY) [37] plus a gamma distribution (G = 0.05), the proportion of invariable sequence (I = 0.21) and estimated transition/transversion bias value (R = 3.81), was selected as the best-fit model to infer phylogeny of the studied population. *Gibbsiella quercinecans* type strain FRB97 was used as the outgroup (S1 Fig). Its corresponding branch was subsequently truncated for better visualisation of very short evolutionary distances between *B*. *goodwinii* strains.

### Clustering methods

The population structure of the clone-corrected dataset with only one representative strain per each of the STs to avoid biased results due to unequal representation, was investigated using discriminant analysis of principal components (DAPC) [38] and STRUCTURE 2.3.4 [39].

### Discriminant analysis of principal components (DAPC)

DAPC is a multivariate technique, implemented in the R package adegenet that makes no assumptions regarding the population model or data structure [40, 38]. It is particularly suited to identifying clusters of genetically related individuals. DAPC uses a sequential K-means procedure followed by assessment of the Bayesian information criterion (BIC), to assess the optimal number of clusters. Cross-validation was used to determine the optimal number of principal components retained in the analysis as previously described [41].

### STRUCTURE analysis

STRUCTURE 2.3.4 [39] was used to group isolates. To estimate the optimal number of clusters, 20 independent runs of K = 1–20 were carried out in STRUCTURE using no priors. Each run had a burn-in of 100,000 iterations followed by 500,000 data-collecting iterations, using a model of correlated allele frequencies and with admixture among populations allowed. CLUMPAK [42] was used to determine the optimal value of K using the ΔK method [43]. CLUMPAK was also used to align all optimum K STRUCTURE runs to the permutation with the highest H-value. The DISTRUCT programme [44] was used to visualize the CLUMPP output.

### F statistics

Arlequin v3.5 [45] was used to calculate pairwise F_st_ values, a measure of population differentiation, and Nm, the predicted number of migrants between populations across the six sampled AOD sites.

### Clonal reproduction and recombination tests

#### Clonal lineages analysis

Lineage assignment and clonal relatedness were analysed using BURST implemented in START2 [36]. A strict method of assignment with bootstrap support was followed, in which 6 out of 7 identical alleles define a lineage.

#### Standardized index of association

Evidence for clonal versus recombining population structure in *B*. *goodwinii* was estimated by assessing the level of linkage between alleles at different loci. To test the null hypothesis, i.e. whether alleles of the seven MLST loci are independent (at linkage equilibrium), the standardized index of association (I^S^_A_) [46] was calculated using START2 (35) with 10,000 iterations. I^S^_A_ values significantly different from 0 (p<0.05) define a clonal population structure (i.e. significant linkage disequilibrium), whereas non-significant values of I^S^_A_ are characteristic of recombining population [47].The test was performed on the entire data set of 44 isolates and also on the clone-corrected dataset.

#### Maximum Chi square test (max *Χ*^*2*^)

The max *Χ*^*2*^ recombination test in START2 [36] was used to detect statistically significant putative recombination events (p<0.05) in individual genes. All possible pairwise comparisons in batch mode for all the genes, were selected for analysis with the maximum number of random trials (n = 10,000).

#### ClonalFrame analysis

The mutation and recombination rates for *B*. *goodwinii* STs were estimated using ClonalFrame v1.1 [48]. The ClonalFrame run consisted of 500,000 MCMC iterations including initial 100,000 burn-in iterations. The parameter ρ/θ estimates the relative frequency of occurrence of recombination and mutation in the phylogenetic history of a species, whereas r/m calculates the relative impact of recombination and mutation in the genetic diversification. The values of ρ/θ and r/m for the clone-corrected dataset were estimated using ClonalFrame GUI by extracting the numbers of mutation events, recombination events, and substitutions introduced by recombination from the ClonalFrame output file.

### Accession numbers for gene sequences used in this study

Gene and corresponding protein sequences were deposited at NCBI Genbank and received the following accession numbers: abc (KY321512-KY321521), dnaN (KY321522-KY321528), dnaJ (KY321529-KY321533), gyrB (KY321534-KY321542), infB (KY321543-KY321546), nusA (KY321547-KY321553) and rpoB (KY321554-KY321560).

## Results

### Gene sequence analysis

Ten housekeeping genes were analysed for the 44 *B*. *goodwinii* strains from the UK. The housekeeping genes LSU, SSU (rDNA) and atpD showed identical sequences across all of the strains and were excluded from further analyses. Seven remaining housekeeping genes, abc, dnaJ, dnaN, gyrB, infB, nusA and rpoB were used to develop and establish the reliable MLST scheme.

The range of intraspecific sequence similarity measured by the percentage of identical sites calculated for each gene showed that the abc gene had the highest level of sequence polymorphism amongst the strains (97.4% identity i.e. 2.6% polymorphisms), followed by dnaN (98.1% identity), gyrB (98.3% identity) and rpoB (98.4% identity). The lowest levels of sequence variation were found for nusA (99.2% identity), dnaJ (99.3% identity), gyrB (99.6% identity), and infB (99.7% identity). The GC content in individual genes exhibited very little variation amongst strains (≤0.5%) with the lowest average in nusA at 52.81% and the highest average in dnaJ (56.84%) (Table 3). The average overall GC content for the concatenated sequence of the seven gene fragments (5997 bp) for all 44 strains was 54.94%.

**Fig 2:**
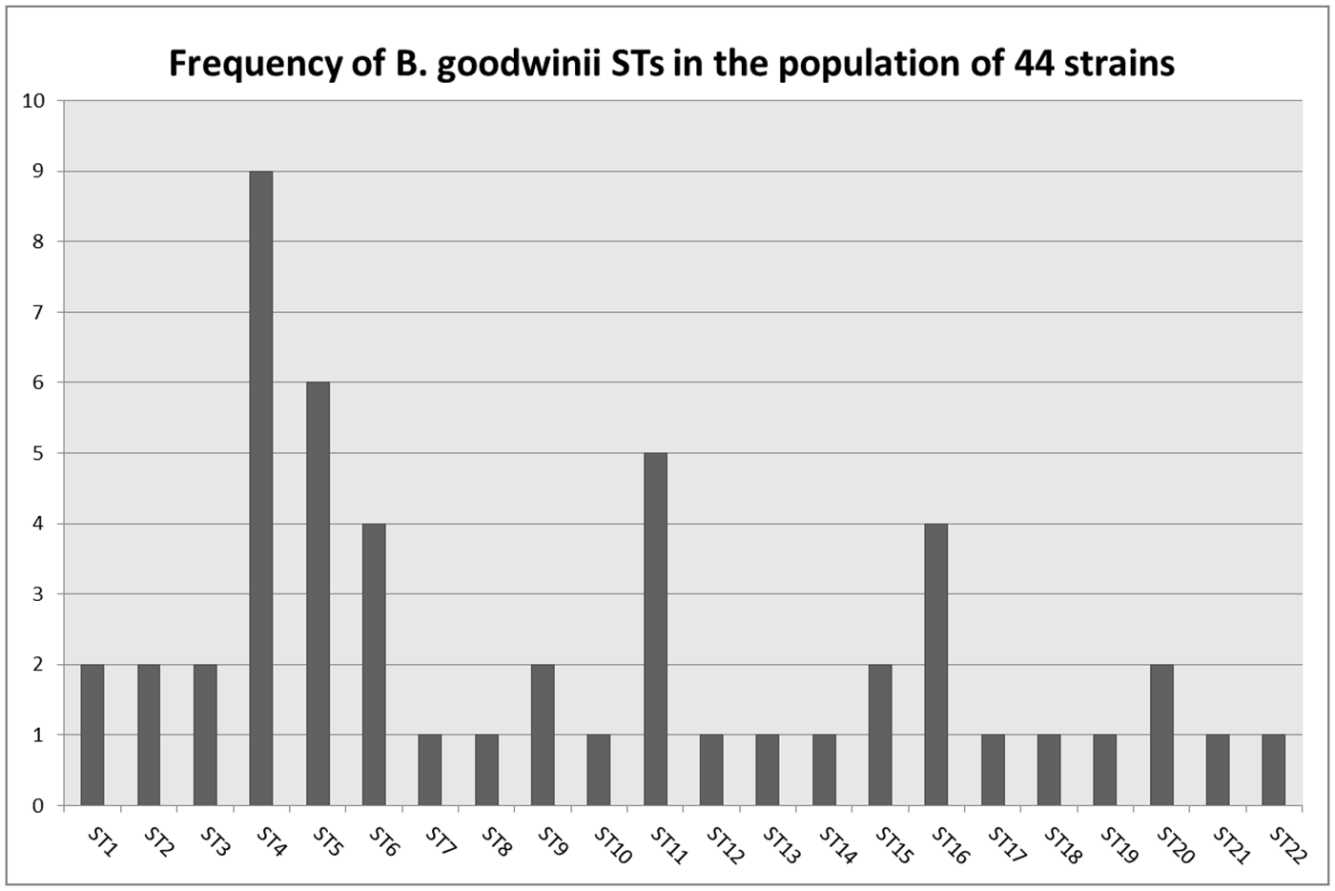
Bar chart showing frequency of STs within the studied population of 44 *B*. *goodwinii* strains. A frequency of ST occurrence is represented on the Y axis.

Twenty two unique sequence types (STs) were found in the 44 *B*. *goodwinii* strains from the 6 study sites, including the *B*. *goodwinii* type strain FRB141 (S1 Table). The overall frequency of occurrence of individual STs ranged from 1 to 10 (Fig 2). The numbers of STs from a specific location varied amongst the sites. Five unique STs were recovered at Attingham Park, four STs each were found at Runs Wood, Bovingdon Hall and Sotterley. Two STs were isolated at Great Monk’s Wood and Bungate Wood. Only one genotype, ST4, was found at two geographically distant sites, i.e. Runs Wood and Bungate Wood. It was also the most frequently isolated ST in the population. All the remaining STs were unique to their geographical location and often to the specific host tree within the site.

The number of alleles at each locus ranged from 4 (infB) to 10 (abc) (S2 Table), and the number of variable base pairs varied between 3 (infB) and 24 (abc) (Table 3). No insertions or deletions were detected within the loci. The nucleotide diversity π-values for individual loci ranged from 0.000838 for infB to 0.010028 for abc (Table 3). Tajima’s D value for the abc locus was significantly different from 0 whereas values for the remaining loci were non-significant (Table 3).

To analyse selection processes amongst the seven MLST genes, the ratio of non-synonymous to synonymous base substitutions (dN/dS) in all allele sequences was determined (Table 3). The results for all the genes showed that non-synonymous sequence polymorphisms were very rare for all the genes (dN/dS < 1).

### Phylogenetic and cluster analyses

#### Maximum likelihood (ML) analysis

**Fig 3:**
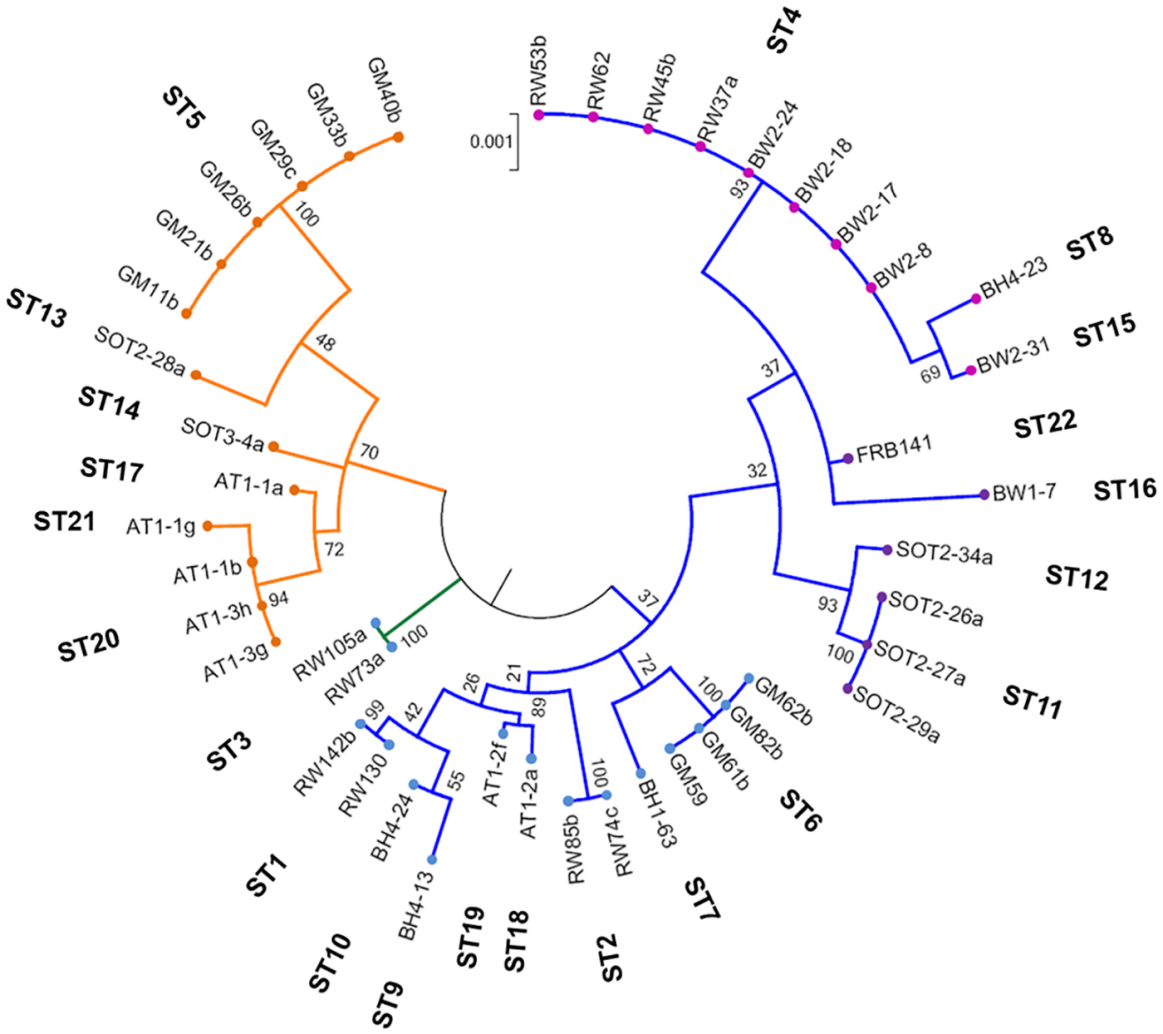
Phylogenetic tree of 44 *B*. *goodwinii* strains based on a concatenated sequence of 7 housekeeping gene fragments (abc, dnaJ, dnaN, gyrB, infB, nusA, rpoB). The evolutionary history was inferred by using the Maximum Likelihood method based on the Hasegawa-Kishino-Yano model (HKY+G+I) *with* estimated transition/transversion bias value. The tree with the highest log likelihood (-13387.9427) is shown. Initial tree for the heuristic search was obtained automatically by applying Neighbor-Join and BioNJ algorithms to a matrix of pairwise distances estimated using the Maximum Composite Likelihood approach, and then selecting the topology with superior log likelihood value. The tree is drawn to scale, with branch lengths measured in the number of substitutions per site. The numbers at the nodes represent the relative distance between the strains with bootstrap support from 1000 resampled datasets. Individual Sequence Type (ST) numbers are indicated. Different colour nodes show the four corresponding clusters estimated by the Discriminant Analysis of Principal Components (Fig 4). Blue and orange colour branches indicate a separation into 2 clusters of STs within the population, whereas green colour branch indicate the recombinant genotype as estimated by STRUCTURE analysis (Fig 5). *Gibbsiella quercinecans* type strain FRB97 (Brady *et al*., 2010) was used as the outgroup (S1 Fig).

Maximum likelihood analysis revealed the presence of two evolutionarily distinct clusters of STs that appeared to have diverged at the root and generated two separate branches of *B*. *goodwinii* isolates within the studied UK population. The bootstrap support value for this delineation was 70% for cluster 1, indicated with orange branches, and 37% for cluster 2 (blue branches) (Fig 3). Similar results were obtained using several other phylogenetic methods, i.e. Minimum Evolution, Neighbor-Joining and UPGMA, which also indicated the presence of the two separate groups of STs (data not shown).

#### Discriminant analysis of principal components (DAPC)

**Fig 4:**
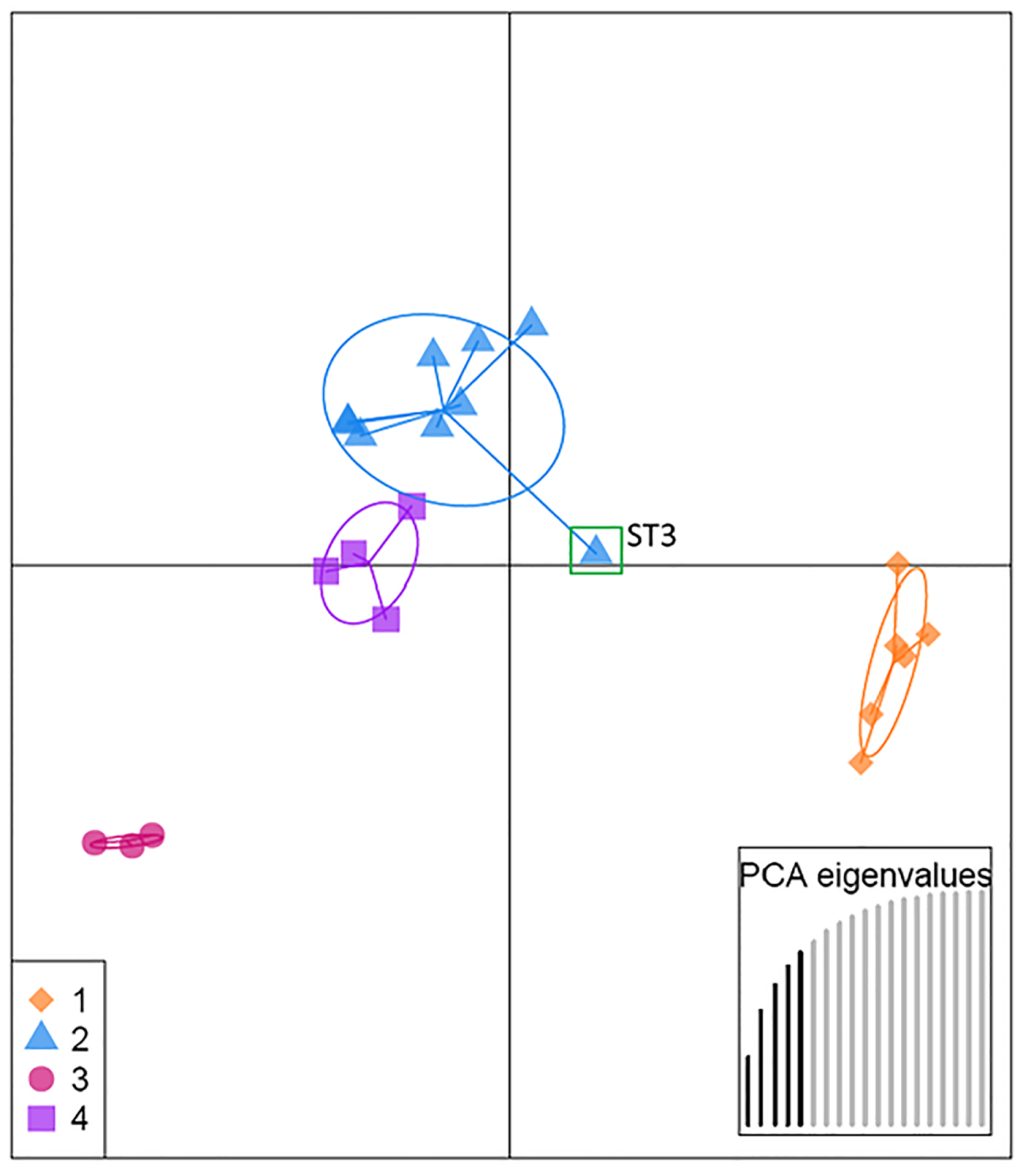
A scatterplot of the discriminant analysis of principal components (DAPC) of *B*. *goodwinii* Sequence Types, showing four clusters of STs. Only the first two principal components of the DAPC are shown (horizontal and vertical axes, respectively). Green rectangle indicates ST3 which is estimated to be an admixed genotype by STRUCTURE with the cluster assignment ratio at 50:50% (Fig 5).

DAPC grouped the STs into 4 distinct clusters (Fig 4). All 4 clusters comprised a mix of STs from several geographical locations. Cluster 1 (orange nodes in Fig 3) consisted of isolates from Attingham Park (ST17, ST20 and ST21), Great Monk’s Wood (ST5) and Sotterley (ST13 and ST14). Cluster 2 (blue nodes in Fig 3) comprised Attingham Park isolates (ST17, ST18, ST19), Bovingdon Hall (ST7, ST9, ST10), Great Monk’s Wood (ST6) and Runs Wood (ST1, ST2, ST3). The smallest number of STs was found in Cluster 3 (pink nodes, Fig 3) and consisted of ST4, which was present at Runs Wood and Bungate Wood, ST16 –found only at Bungate Wood, and ST8 present only at Bovingdon Hall (ST8). Cluster 4 (violet nodes, Fig 3) was formed from STs from Bungate Wood (ST16), Sotterley (ST11, ST12), and also included *B*. *goodwinii* type strain FRB97(T) from Outwood (ST22). Clusters 2 and 4 plotted close together, whereas the remaining clusters 1 and 3 were placed far apart and appeared more distant to any other cluster in the analysis (Fig 4). There was no apparent correlation or trend between clusters and their geographical distribution.

#### STRUCTURE analysis

**Fig 5:**
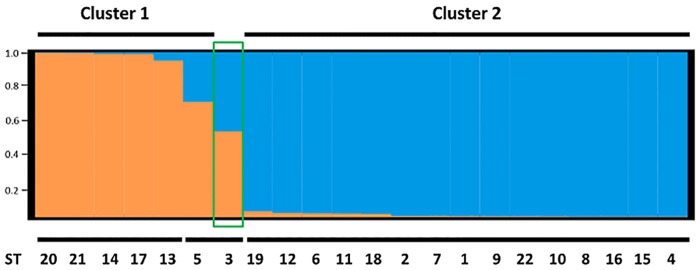
Bayesian clustering of *B*. *goodwinii* STs inferred using the programme STRUCTURE. Vertical columns represent individual STs, labelled below the figure, whose membership probabilities to the two clusters are represented by orange and blue sections of the column.

STRUCTURE analysis suggested that two clusters best describe the data (best K = 2), cluster 1 (orange) and 2 (blue) (Fig 5). Cluster 2 appears to form a genetically homogeneous group of STs, whereas cluster 1 is genetically heterogeneous with genetic material presumably acquired from cluster 2. ST3 and ST5 showed a high level of admixture (cluster assignment probability ratios of approximately 0.5: 0.5 and 0.7: 0.3, respectively). For the remaining STs, cluster admixture was negligible (i.e. <0.05 assignment probability to the other cluster).

### F statistics and migration

The highest F_st_ value (F_st_ = 0.68909) was observed between Bungate Wood and Attingham Park populations (S3 Table). The lowest F_st_ values were calculated for RunsWood and Bovingdon Hall (F_st_ = 0.01717), and Runs Wood and Bungate Wood (F_st_ = 0.1853) populations. The estimated number of migrants, Nm, was highest (28.61265) between Runs Wood and Bovingdon Hall (S3 Table), also confirming a close relationship of the populations from these locations and indicating a potential migration pattern. The second highest Nm value of 2.19831 was observed for Runs Wood and Bungate Wood subpopulations both of which were dominated by the ST4 genotype. It was also the only ST among the studied UK *B*. *goodwinii* population that was found at two geographically distant locations.

### Clonal reproduction versus recombination

#### Lineage assignment (BURST)

The BURST method was used to assign strains to clonal lineages. Five clonal groups and 10 singleton lineages were detected amongst all 22 STs. Group 1 consisted of ST9, ST10 and ST16 with the predicted founder of the lineage being ST9. Group 2 (ST11, ST12 and ST13) had multiple predicted founder candidates (either of these STs). Group 3 (ST18 and ST19), Group 4 (ST4 and ST15) and Group 5 (ST20 and ST21) had no predicted founder. Each of the remaining STs represented an individual unique clonal lineage.

#### Standardized index of association (I^S^_A_)

In order to understand whether the *B*. *goodwinii* population was clonal or recombining, a standardized index of association (I^S^_A_) was calculated [45]. The analysis of all 44 isolates yielded an I^S^_A_ value of 0.2122 (p≤0.0001), that showed significant linkage disequilibrium, indicating the persistence of clonally expanding STs. I^S^_A_ values were also calculated separately for subpopulations from each geographical location. In all subpopulations significant linkage disequilibrium was detected showing that clonal reproduction was maintained even at short distances, i.e. for a local subpopulation of different STs that may come into direct contact with each other (STs from the same host tree/ AOD lesion). The I^S^_A_ values for Runs Wood, Great Monk’s Wood, Bovingdon Hall, Sotterley, Bungate Wood and Attingham were 0.598, 0.7879, 0.3175, 1.1155, 0.791 and 0.3631, respectively (p≤0.0001 for all). When only one representative strain of each ST was included in the analysis, the overall I^S^_A_ value decreased to 0.087, but remained significant (p≤0.0001).

#### Max Chi square test (max *Χ*^*2*^)

In addition to the I^S^_A_ analysis that showed a clonal mode of reproduction amongst lineages and STs, max *Χ*^*2*^ test was carried out to study if recombination could have also played any role in shaping the observed population structure of *B*. *goodwinii* in the UK. Four significant putative recombination events (P<0.05) were detected in gyrB locus. The highest max *Χ*^*2*^ value of 45.772 was obtained for gyrB-1 (ST7) and gyrB-7 (ST17, ST19, ST20) alleles. The remaining 3 significant putative recombination events occurred between gyrB-8 (ST2) and gyrB-9 (ST4) (max *Χ*^*2*^ = 42.4769), gyrB-6 (ST6) and gyrB-7 (ST17, ST19, ST20) (max *Χ*^*2*^ = 40.9942) and finally gyrB-1 (ST7) and gyrB-6 (ST6) (max *Χ*^*2*^ = 40.9942).

#### ClonalFrame

Results from ClonalFrame analysis suggested that mutations have occurred twice as frequently as recombination events (ρ/θ ≈ 0.49 with 95% confidence interval of 0.158–1.12). However, the statistic r/m ≈ 1.5 (with 95% confidence interval of 0.52–3.19) demonstrated that recombination has had a considerable effect on the evolution of *B*. *goodwinii* in the UK, i.e. despite being less frequent than mutation, recombination events have introduced almost 1.5 times more substitutions than random mutation.

## Discussion

Here we report the development and use of a MLST scheme to determine the genetic variability and population genetic structure of *B*. *goodwinii* strains isolated from native oak expressing symptoms of AOD at different locations in the UK. To our knowledge this is the first population genetic analysis of this species.

The MLST method proved well suited for characterisation of the diversity and population structure of *B*. *goodwinii* in Britain. Changes in protein sequences were rare, indicated by very low values of non-synonymous to synonymous base substitutions ratios of the housekeeping gene sequences (dN/dS< 1) (Table 3), and demonstrated a purifying selection against changes in amino acid composition. This showed that sequence variability is selectively neutral at the protein level, and that the selected housekeeping genes were good candidates for the development of a MLST scheme. However, the relatively high number of polymorphisms identified in the abc gene sequences compared with other genes could also indicate that balancing selection acts upon this locus, which could also be supported by the significantly positive Tajima’s D values. It could also indicate a rapid decrease in population size, balancing selection [49], or subdivision of the population [50]. Furthermore, it could also mean that the observed differences in the abc gene among individuals are caused by both purifying selection, which limits the amino acid changes due to functional restrictions of this gene, and potential balancing selection, which maintains the genetic polymorphism among *B*. *goodwinii* strains. Tajima’s D values for the remaining loci were non-significant, which demonstrated that these genes evolved neutrally, i.e. evolution by random processes [35]. Individually, none of the seven MLST loci provided resolution that allowed identification of all the STs in the studied population, justifying the use of a multilocus approach for resolving the exact phylogeny (S1 and S2 Tables).

The population structure of *B*. *goodwinii* was elucidated by the correlative use of several approaches. The result obtained using the MLST method showed that strains in the UK separated into 22 different STs. Such a level of intraspecific diversity across a relatively small set of strains used in the study could suggest that there could be many more STs of *B*. *goodwinii*. Despite the low sample size, the analysis demonstrated that the bacterial population in the UK exhibits high variation and these results therefore provide meaningful initial insights into the population structure of *B*. *goodwinii*.

The ML phylogenetic inference method and statistical clustering methods, i.e. STRUCTURE and DAPC analyses indicated the presence of phylogenetically distinct clusters of individuals in the population. The ML analysis provided a 70% bootstrap support for the presence of cluster 1, indicated by orange branches (Fig 3), but only 37% support (therefore no significant support) for cluster 2 (blue branches; Fig 3) within *B*. *goodwinii* STs, thus confirming that all isolates tested belonged to the same taxon. However, the two clusters indicated by ML which delineate intraspecific groupings are still relevant as STRUCTURE analysis also showed two similar distinct clusters among individuals. The low support values could be due to recombination that occurred in some loci resulting in placement of STs in different groups, which could consequently affect significance of bootstrap support. To further support the presence of the two clusters of STs in the population, several additional phylogenetic analyses were carried out using Minimum Evolution, UPGMA and Neighbour-Joining methods, which also suggested the existence of the two clusters (data not shown). On the other hand, DAPC analysis indicated four clusters of STs that correlated with a further branching of the two clusters into two lineages (Figs 3 and 4). Interestingly, ML analysis also showed the presence of an intermediate ST (ST3 indicated by green branch; Fig 3) which was also assigned by STRUCTURE to both clusters at approximately 50:50 ratio (Fig 5) and thus was considered an admixed ST. In DAPC, although ST3 was assigned to cluster 2 (Fig 4; blue), it was an outlier, placed between cluster 2 and cluster 1 (orange; Fig 4).

Generally, STs tended to be unique to their place of origin however, the distribution of closely related lineages and clusters appeared unrelated to the geographic location or year of isolation. Only one genotype (ST4) was identified at two distant locations (Bungate Wood and Runs Wood) indicating a recent migration of this ST. However, it was not possible to determine the direction in which the spread of clones could have occurred. For the populations of Runs Wood and Bungate Wood, both of which were dominated by ST4, as well as between Runs Wood and Bovingdon Hall subpopulations, F_st_ values were low (<0.18) and Nm values high (>2.19). F_st_ values between all remaining sites were considerably higher, suggesting a high degree of genetic differentiation but negligible gene flow. Both phylogenetic and genetic differentiation analyses suggest that the STs found at the individual locations are genetically isolated but are not separate clonal lineages that have been locked in place by geographical barriers. This is also supported by low M values obtained in Migration analysis. One possible explanation for the lack of association between lineages and their geographic origin, i.e. genetically similar populations occur in geographically distinct areas, may be the transportation of infected wood and/or equipment between woodland sites. Yet another explanation could be the migration of TSOB, the native beetle that is closely associated with AOD outbreaks, which may have carried and spread the bacteria between sites.

Depending on the mode of evolutionary processes that shape the population structure, two types of bacterial populations can be distinguished. Populations of a species in which individuals vary mainly because of the occurrence of random mutations are defined as clonal whereas bacterial populations in which diversity is mainly generated by recombination are defined as panmictic, i.e. freely recombining [51]. Identification of the relative effects of mutation and recombination in bacterial population allows further classification of the pathogenic species into two main groups. In the epidemic population structure, individual members may initially recombine and expand rapidly to become the founder individuals for a new clonal population of pathogenically better fit strains [52, 53]. Subsequently, all the diseased individuals during an epidemic are colonized by the clonal population of the pathogen. In an endemic population structure, a pathogen that emerged a relatively long time ago has diverged into many different clones. The individual hosts are colonized by a number of diverse clones and therefore recombination between them at some frequency is likely (50). However, linkage disequilibrium (the non-random association of alleles) may still be observed in a sample of strains even if the population from which the sample was drawn is recombining at a high frequency [53, 54]. Significant linkage disequilibrium maintained between *B*. *goodwinii* MLST alleles suggests a non-random distribution of alleles and a clonal population structure where diversity increases by the accumulation of point mutations. Furthermore, BURST analysis allowed identification of 15 clonal lineages among the STs, of which 5 were clonal groups and the remaining 10 were singletons. Although linkage disequilibrium remained significant, the low I^S^_A_ values, especially for the clone-corrected dataset, could also suggest that recombination may still occur between individuals. In fact, Max *Χ*^*2*^ recombination test showed putative recombination events however they were statistically significant (P < 0.05) only for gyrB alleles. STRUCTURE analysis also suggested that recombination events have occurred between the clusters. The observed genetic exchange and recombination across the two ancient lineages does not support a strictly clonal population structure. The relative effect of mutation and recombination processes on the observed diversity in a set of strains that belong to a given bacterial species varies from species to species. Despite the apparent spontaneous mutation that seems to drive the population diversity in *B*. *goodwinii*, recombination could still be taking place, but for such events to break linkage disequilibrium between the alleles, a given allele must change a minimum of 20 times more frequently by recombination than by spontaneous point mutation to achieve random assortment within a bacterial population [52]. Results from STRUCTURE analysis suggest that only ancestral cluster 1 seems to exhibit some level of mixed ancestries suggesting that limited, unidirectional interlineage admixture has taken place in the past (Fig 5). Such recombination between ancestral clusters is also consistent with our results from the phylogenetic analysis (Fig 3), indicating divergence of the two clusters. The frequency of occurrence of recombination versus mutation (ρ/θ ≈ 0.49), and the relative effect of recombination versus mutation in genetic diversification (r/m ≈ 1.5) suggest that the recombination rate in the UK population of *B*. *goodwinii* appears to be maintained at moderate levels. Therefore, it is suggested here that the *B*. *goodwinii* population on AOD-affected oak in Britain exhibits an endemic population structure in which the individual hosts and sites appear to be colonized by many different clonal lineages and therefore recombination between them at some frequency is likely. Since our analysis indicates that the clustering of STs is not caused by geography, the barrier that may be stopping interlineage gene transfer and movement of genotypes between the sites could be caused by some other, so far undetermined factors. One possible explanation could be the relatively recent nature of the AOD outbreak in Britain. *B*. *goodwinii* was first described as a species less than five years ago [8]. Therefore, strains that were available for our study were isolated within the past five years, making the existent culture collection limited and obtaining much older strains is not possible due to the lack of suitable preserved older oak material. It is therefore not possible to substantiate beyond any doubt if the observed lack of geographic clustering could be due to a previous expansion of *B*. *goodwinii* and a subsequent lack of mixing between the sites. Consequently, this could have driven the more recent differentiation, masking hypothetical initial pattern of the distribution of the observed clusters. On the other hand, the opposite scenario where the strains are expanding rapidly and transfer between sites appears to be common in bacteria, which could also make it difficult to identify and interpret any patterns of geographical migration.

The use of limited set of strains in this study was largely due to lack of access to bark material from land owners as sampling method is a damaging process and thus could potentially affect the wood quality for the future timber harvest as well as decrease the aesthetic value that veteran oak trees carry in parkland areas. However, despite a low number of strains, the MLST scheme developed in this study revealed high variation in *B*. *goodwinii* and it could therefore be conceivable that many more unique STs yet to be described could be present in the UK population.

AOD is a relatively new and complex disease that occurs in the UK, and several bacterial species associated with AOD lesions have recently been reported. The MLST method developed in this study allowed the investigation of the diversity and population structure of one of the most frequently associated species—*B*. *goodwinii*. The structure of its population has now been characterised and the MLST scheme developed here has established a database that can be accessed by any laboratory around the world. This will be particularly useful for identifying any strains that spread to new geographical locations and/or host and should allow a better understanding of the biotic factors contributing to the oak decline syndrome in the UK and worldwide.

## Supporting information

S1 Fig Phylogenetic analysis of intraspecific variation by maximum likelihood method among *B*. *goodwinii* strains used in this study. Closely related Enterobacterium *Gibbsiella quercinecans* type strain FRB97 was used as outgroup. Due to very short evolutionary distances, only subtree comprising *B*. *goodwinii* strains are presented in the main body of the manuscript (Fig 3).
(DOCX)

S1 Table Allelic profiles of the 22 *B*. *goodwinii* STs identified in this study. Only one strain per ST is presented.
(DOCX)

S2 Table Allele frequencies for seven housekeeping genes analysed in this study. (DOCX)

S3 Table A matrix of Fst values (bold font) and corresponding number of migrants (Nm). (DOCX)
