# Supplementary material for: Multilocus sequence typing provides insights into the population structure and evolutionary potential of Brenneria goodwinii, associated with acute oak decline
Source: PLoS One. 2017 Jun 1;12(6):e0178390. doi: 10.1371/journal.pone.0178390 (PMC5453491; doi:10.1371/journal.pone.0178390)
Supplement: S1 Fig — Closely related Enterobacterium Gibbsiella quercinecans type strain FRB97 was used as outgroup. Due to very short evolutionary distances, only subtree comprising B. goodwinii strains are presented in the main body of the manuscript (Fig 3). (DOCX) [file pone.0178390.s001.docx]

**S1 Fig.**

**Phylogenetic analysis of intraspecific variation by Maximum Likelihood method among *B. goodwinii* strains used in this study*.*** Closely related Enterobacterium *Gibbsiella quercinecans* type strain FRB97 was used as outgroup. Due to very short evolutionary distances, only subtree comprising *B. goodwinii* strains are presented in the main body of the manuscript (Fig. 3).
