# Supplementary material for: Multilocus sequence typing provides insights into the population structure and evolutionary potential of Brenneria goodwinii, associated with acute oak decline
Source: PLoS One. 2017 Jun 1;12(6):e0178390. doi: 10.1371/journal.pone.0178390 (PMC5453491; doi:10.1371/journal.pone.0178390)
Supplement: S1 Table — Only one strain per ST is presented. (DOCX) [file pone.0178390.s002.docx]

**S1 Table. Allelic profiles of the 22 *B. goodwinii* STs identified in this study.** Only one strain per ST is presented.

| Strain | ST | *abc* | *dnaJ* | *dnaN* | *gyrB* | *infB* | *nusA* | *rpoB* |
| --- | --- | --- | --- | --- | --- | --- | --- | --- |
| RW130 | **1** | 9 | 3 | 3 | 4 | 4 | 2 | 7 |
| RW74c | **2** | 5 | 5 | 2 | 8 | 4 | 6 | 7 |
| RW73a | **3** | 4 | 5 | 5 | 5 | 4 | 5 | 2 |
| RW62 | **4** | 10 | 5 | 7 | 9 | 1 | 5 | 1 |
| GM11b | **5** | 3 | 5 | 7 | 9 | 4 | 1 | 6 |
| GM59 | **6** | 8 | 5 | 1 | 6 | 4 | 3 | 7 |
| BH1-63 | **7** | 10 | 5 | 1 | 1 | 4 | 7 | 4 |
| BH4-23 | **8** | 9 | 5 | 7 | 5 | 1 | 5 | 1 |
| BH4-13 | **9** | 5 | 5 | 3 | 5 | 4 | 3 | 6 |
| BH4-24 | **10** | 9 | 5 | 3 | 5 | 4 | 3 | 6 |
| SOT2-26 | **11** | 9 | 2 | 6 | 9 | 4 | 7 | 5 |
| SOT2-34a | **12** | 5 | 2 | 6 | 9 | 4 | 7 | 5 |
| SOT2-28a | **13** | 3 | 2 | 6 | 9 | 4 | 7 | 5 |
| SOT3-4a | **14** | 2 | 1 | 4 | 3 | 4 | 4 | 7 |
| BW2-31 | **15** | 10 | 5 | 7 | 2 | 1 | 5 | 1 |
| BW1-7 | **16** | 5 | 5 | 7 | 5 | 4 | 3 | 6 |
| AT1-1a | **17** | 1 | 5 | 4 | 7 | 4 | 7 | 7 |
| AT1-2a | **18** | 6 | 5 | 3 | 2 | 4 | 7 | 7 |
| AT1-2f | **19** | 6 | 5 | 3 | 7 | 4 | 7 | 7 |
| AT1-1b | **20** | 1 | 5 | 4 | 7 | 3 | 7 | 3 |
| AT1-1g | **21** | 1 | 5 | 4 | 2 | 3 | 7 | 3 |
| FRB141 | **22** | 7 | 4 | 7 | 9 | 2 | 6 | 6 |
