# Supplementary material for: Multilocus sequence typing provides insights into the population structure and evolutionary potential of Brenneria goodwinii, associated with acute oak decline
Source: PLoS One. 2017 Jun 1;12(6):e0178390. doi: 10.1371/journal.pone.0178390 (PMC5453491; doi:10.1371/journal.pone.0178390)
Supplement: S2 Table — (DOCX) [file pone.0178390.s003.docx]

**S2 Table. Allele frequencies for seven housekeeping genes analysed in this study.**

| Allele | abc | dnaJ | dnaN | gyrB | infB | nusA | rpoB |
| --- | --- | --- | --- | --- | --- | --- | --- |
| 1 | 5 | 1 | 5 | 1 | 10 | 6 | 10 |
| 2 | 1 | 5 | 2 | 3 | 1 | 2 | 2 |
| 3 | 7 | 2 | 6 | 1 | 4 | 7 | 4 |
| 4 | 2 | 1 | 6 | 2 | 29 | 1 | 1 |
| 5 | 5 | 35 | 2 | 6 | - | 12 | 5 |
| 6 | 2 | - | 5 | 4 | - | 3 | 10 |
| 7 | 1 | - | 18 | 5 | - | 13 | 12 |
| 8 | 4 | - | - | 2 | - | - | - |
| 9 | 7 | - | - | 20 | - | - | - |
| 10 | 10 | - | - | - | - | - | - |
| Unique | 10 | 5 | 7 | 9 | 4 | 7 | 7 |
