# Supplementary material for: Multilocus sequence typing provides insights into the population structure and evolutionary potential of Brenneria goodwinii, associated with acute oak decline
Source: PLoS One. 2017 Jun 1;12(6):e0178390. doi: 10.1371/journal.pone.0178390 (PMC5453491; doi:10.1371/journal.pone.0178390)
Supplement: S3 Table — (DOCX) [file pone.0178390.s004.docx]

**S3 Table. A matrix of F statistics (F_st_) (bold font) and corresponding number of migrants (Nm) values.**

| F_st_ \ Nm | AT | BH | BW | GM | RW | SOT |
| --- | --- | --- | --- | --- | --- | --- |
| AT | * | 0.62832 | 0.2256 | 0.79967 | 0.83011 | 0.67013 |
| BH | **0.44314** | * | 0.75498 | 0.76384 | 28.61265 | 0.8423 |
| BW | **0.68909** | **0.39841** | * | 0.48688 | 2.19831 | 0.43257 |
| GM | **0.38471** | **0.39562** | **0.50665** | * | 0.95588 | 0.8368 |
| RW | **0.37591** | **0.01717** | **0.1853** | **0.34343** | * | 1.3769 |
| SOT | **0.4273** | **0.37249** | **0.53615** | **0.37403** | **0.2664** | * |
